# Development of Biodegradable Cosmetic Patch Using a Polylactic Acid/Phycocyanin–Alginate Composite

**DOI:** 10.3390/polym12081669

**Published:** 2020-07-27

**Authors:** Sarah Amalina Adli, Fathilah Ali, Azlin Suhaida Azmi, Hazleen Anuar, Nur Aimi Mohd Nasir, Rosnani Hasham, Mohamad Khairul Hafiz Idris

**Affiliations:** 1 Department of Biotechnology Engineering, Kulliyyah of Engineering, International Islamic University Malaysia, Jalan Gombak, Kuala Lumpur 53100, Malaysia; salegacyjr@gmail.com (S.A.A.); azlinsu76@iium.edu.my (A.S.A.); 2 Department of Manufacturing and Materials Engineering, Kulliyyah of Engineering, International Islamic University Malaysia, Kuala Lumpur 53100, Malaysia; hazleen@iium.edu.my (H.A.); aimi_nasir@ymail.com (N.A.M.N.); 3 Institute of Bioproduct Development, University Teknologi Malaysia, Johor Bahru 81310, Johor, Malaysia; r-rosnani@utm.my (R.H.); mhkhairul@gmail.com (M.K.H.I.)

**Keywords:** patches, cosmetic, phycocyanin, PLA, spirulina extract, alginate

## Abstract

The usage of non-degradable polymer as the main matrix for a cosmetic patch raises concern, as it can cause environmental pollution when discarded in landfill. Thus, biodegradable polylactic acid (PLA) was chosen in this study, as PLA has non-toxic properties and similar mechanical properties to conventional plastic materials. An active ingredient in a cosmetic patch serves the purpose of providing beneficial ingredients to the skin; therefore, phycocyanin, an extract from spirulina, was chosen, as it possesses antioxidant and anti-inflammatory properties. Alginate was also incorporated with the phycocyanin for fabrication onto the PLA matrix. A preliminary study was first carried out to identify the antioxidant properties and cytotoxic effect of the phycocyanin on skin cells. It was observed that phycocyanin had no cytotoxic effect on the skin and showed good antioxidant activity. PLA/phycocyanin–alginate composite was fabricated using a solvent casting method, and optimization of preparation conditions (phycocyanin/alginate ratio, stirring time, and temperature) were carried out using the one-factor-at-a-time (OFAT) method with responses of elongation at break and releasing properties. Attenuated total reflectance (ATR)–FTIR analysis was also conducted to further analyze the functional group of the composites. Surface morphologies were observed for samples before and after the releasing test. From the analyses conducted, PLA/phycocyanin–alginate composite prepared at a phycocyanin/alginate ratio of 40/60 for 20 h at 20 °C gave the best properties in terms of flexibility of film and releasing properties of phycocyanin.

## 1. Introduction

In the past, patches were usually made of cotton wool or bandages to protect the targeted area from the surroundings. However, in the usage of cotton, the outer layer is moistened to activate the patch, causing the patch to be vulnerable to bacterial contamination [1]. Other than that, when cotton wool or gauze is used as a patch, it will lose moisture with time, and this can cause discomfort on the skin when removing it [2].

Patches have a high demand for their versatility of applications such as cosmetics [3], dermatitis [4], and drug delivery [5]. As patches function by delivering active compounds or medication, they must possess adhesive material and properties for the patches to work within the desired span of time [6]. Other than that, the patches need to be fabricated with active ingredients that will give a beneficial effect to the skin, such as an antioxidant property and an anti-inflammatory effect [7].

Today, release efficiency of active ingredients or drugs is vigorously being researched for consumers’ benefit. Patches are usually made of polymer to support their matrix where the active ingredients are embedded. Therefore, various methods have been explored, such as the electrospinning method [8], microneedle array method [9], etc., for active ingredients to be released from polymer matrices.

Polylactic acid (PLA), a biodegradable polymer, stands out for this purpose compared to other types of biodegradable polymers. As PLA is produced from an agricultural crop, it is deemed safe by the FDA [10]. PLA is also distinctive from other polymers as it degrades naturally into the environment and possesses similar properties to conventional plastics, such as polystyrene and polyethylene. PLA has been researched extensively for packaging materials [11,12], and it can be explored further as a matrix for cosmetic patches.

As mentioned previously, the polymer used for patches needs to be impregnated with active ingredients for it to be beneficial to the skin. The active ingredients can be from many types of sources such as fruits, plants, and many more, as long as they possess active ingredients that are good for skin. Many researchers have used spirulina as their active ingredient for application in both cosmetic patches and medical patches, as spirulina contains many kinds of bioactive compounds that work as antioxidants and have anti-inflammatory effects [6,13]. Hence, in this study, a patch comprising PLA as a matrix and spirulina extract (phycocyanin) as the active ingredient was developed based on its benefits.

## 2. Materials and Methods

### 2.1. Materials

Polylactic acid (Ingeo 3251D) was purchased from NatureWorks (Minnetonka, MN, USA) with an average molecular weight (Mw) of 148,000 g/mol. Phycocyanin powder was purchased from Xi’an Quanao Biotech Co., Ltd. (Xi’an, China). Alginate powder was purchased from Take it Global company. Chloroform was purchased from HmbG chemicals (analytical grades, Hamburg, Germany) and used as is. A preliminary study, a cell cytotoxicity test, was first done on the raw material phycocyanin to evaluate the cytotoxic effect of raw material on skin cells. The test was carried out by a 3-(4,5-dimethylthiazol-2-yl)-2,5-diphenyltetrazolium bromide (MTT) assay with a human skin fibroblast cell line, HSF1184.

### 2.2. Preparation of PLA/Phycocyanin–Alginate Composites

**Figure 1:**
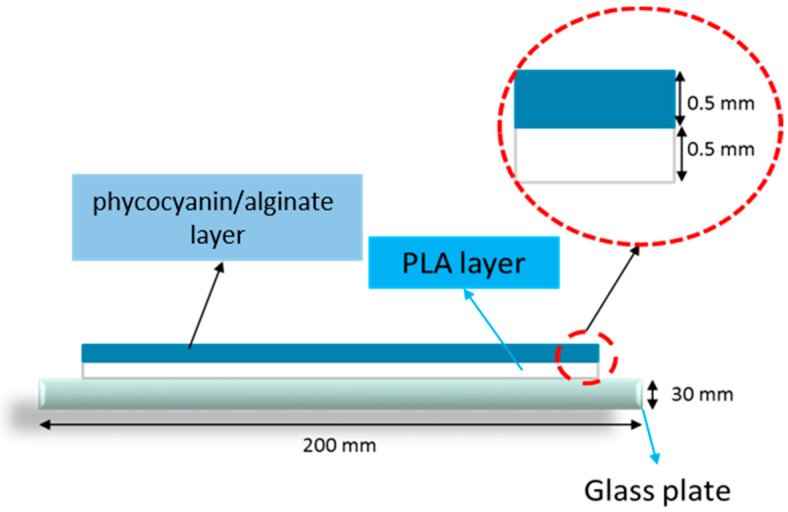
Schematic diagram of two layers of the composite.

A PLA/phycocyanin–alginate composite patch was fabricated using a solvent casting method. Firstly, PLA beads and algae powder were oven-dried for 24 h at a temperature of 40 °C before use. PLA was then dissolved in chloroform for four hours. After the PLA was dissolved, the polymer solution was then poured onto a clean glass plate and left to dry for about three hours. Secondly, a mixture of phycocyanin and alginate powder (ratio 0.5:0.5) was mixed in 40 mL distilled water before being poured onto the PLA film on the glass plate. This PLA/phycocyanin–alginate film was then left to dry for 24 h. Figure 1 shows the schematic diagram of a PLA/phycocyanin–alginate composite, consisting of two layers.

PLA/phycocyanin–alginate composites were prepared by optimizing the ratio of phycocyanin/alginate, stirring time, and temperature using the one-factor-at-a-time (OFAT) method. The ratio of phycocyanin was conducted in the range of 10 to 50 wt%, the temperature range was from 25 to 45 °C, and the mixing time range was from 4 to 24 h (samples prepared at 4 and 8 h were eliminated because samples formed were non-homogeneous). The responses recorded were elongation at break (response 1) and absorbance optical density (OD) (response 2). Elongation at break was selected as the first response, as flexibility is an important factor for a patch for its durability [14], whereas for absorbance as the second response, it was selected because a rapid release of the bioactive compound is desired in a cosmetic patch [15].

### 2.3. Characterizations

#### 2.3.1. Antioxidant Assay

A 1,1′-diphenyl-2-picrylhydrazyl (DPPH) radical scavenging activity was conducted to evaluate the antioxidant activity of phycocyanin. The test was conducted using a DPPH microplate protocol in a 96-well plate. A total of 100 µL of stock solution was first added to a 96-well plate, followed by 100 µL of DPPH and methanol mixture (19.7 mg DPPH powder dissolved in 250 mL methanol). The microplate was wrapped with an aluminum foil and left for 30 min. The absorbance value of each reaction was taken at 517 nm. The measurements were carried out in triplicate. DPPH scavenging activity was calculated using the following formula:

(1) $$\%\;\mathrm{DPPH}\;\mathrm{scavenging}=\left[\frac{{\left(A\right)}_{control}-{\left(A\right)}_{sample}}{{\left(A\right)}_{control}}\right]\times 100,$$

#### 2.3.2. Cell Cytotoxicity Analysis

A cell cytotoxicity test was conducted to evaluate the phycocyanin on skin cells. The test was carried out by a 3-(4,5-dimethylthiazol-2-yl)-2,5-diphenyltetrazolium bromide (MTT) assay with a human skin fibroblast cell line, HSF1184.

- Cell culture

Human skin fibroblast cells (HSF 1184) were purchased from American Type Culture Collection (ATCC), Rockville, MD, USA. The cells were maintained in Dulbecco’s Modified Eagle’s Medium (DMEM) supplemented by 10% fetal bovine serum (FBS) and 1% penicillin–streptomycin (PS). Incubation was carried out in a 5% CO2 incubator at 37 °C. For experiment, the cells from passage 6–8 were used.

- Phycocyanin treatment

Human skin fibroblast cells were seeded at a density of 1 × 10^4^ cells/well in 96-well culture dishes and cultured in DMEM supplemented by 10% FBS and 1% PS to 80% confluence. Cells were then starved in serum free DMEM for 24 h and rinsed with phosphate-buffered saline (PBS). The cells were then cultured in serum free DMEM with or without phycocyanin for 48 h. For every experiment, phycocyanin was dissolved in distilled water.

- MTT assay

To examine the effect of phycocyanin on the cell cytotoxicity effect, a Thiazolyl Blue Tetrazolium Blue (MTT) assay was performed. Following treatment with phycocyanin, the culture medium was removed and 50 µl of the MTT solution (0.2 mg/mL) was added for four hours at 37 °C. The solution was then removed and 200 µL DMSO was added to dissolve the formazan crystal. The optical density (OD) was then taken at 570 nm using a microplate reader (BioTek ELx808).

#### 2.3.3. Materials Characterizations

The elongation at break of polymer composites was determined using the Universal Testing Machine (Shimadzu, AGS-X, Kyoto, Japan). Samples were prepared according to American Society for Testing and Materials (ASTM) D-882 type-V with crosshead speed 5 mm/min, load cell of 5 kN, and 30 mm gauge length. The results were taken as an average of 5 tests. Morphology of the composites was investigated using Scanning Electron Microscopy (JEOL, JSM-5600, Welwyn Garden, UK) at 8 and 12 kV accelerating voltages and coated with palladium sputter before the analysis. For the releasing test, the patches were cut into 1 × 1 cm pieces (16 pieces in total) and then were placed in a 24-well plate. Distilled water was then poured into each well and left for 30 min before 200 µl were aliquoted from 24 wells to 96 wells. The absorbance was recorded at 620 nm. Functional groups in PLA, phycocyanin, alginate, and PLA/phycocyanin–alginate prepared in various optimizing conditions were determined using an attenuated total reflectance Perkin Elmer Spectrum 1000 series (USA). A total of 16 scans were done on the samples, and spectra were collected between 4000 and 400 cm^−1^ wavenumber range at 4 cm resolution.

## 3. Results and Discussion

### 3.1. Preliminary Test (Cell Cytotoxicity Test and Antioxidant Activity)

#### 3.1.1. Cell Cytotoxicity Test

**Figure 2:**
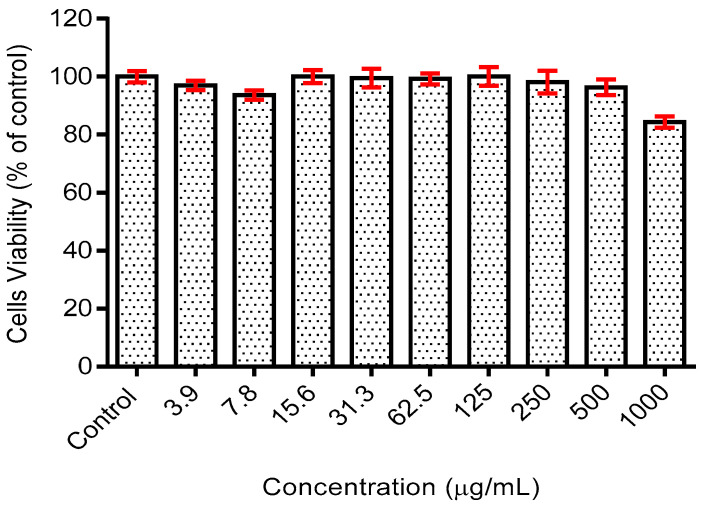
Cytotoxicity effects of phycocyanin on the HSF1184 cell.

During preliminary study, a cytotoxicity test of the phycocyanin was conducted on the human fibroblast skin cell line, HSF1184, to verify and develop the toxicity profile of the compound on the healthy skin cell line. Within the tested concentration (3.9–1000 µg/mL), the extract exhibited minimal toxicity on the cell line with viability greater than 80% in comparison to the control group (untreated) for all doses tested (Figure 2). Moreover, the half maximal inhibition concentration (IC_50_) analysis using statistical software (GraphPad Prism V.6) resulted in an IC_50_ value of >1000 µg/mL, precisely at 2216 µg/mL. The low toxicity profile of the phycocyanin could be due to its protective potential. It was reported that treatment with phycocyanin at a low dose (0–250 µg/mL) had been shown to exhibit a protective effect against oxidative-induced cell death [16]. The presence of phycocyanin upregulated the expression of factors responsible for the growth of fibroblast-keratinocyte co-culture, including transforming growth factor (TGF)-α1 and interleukin (IL)-1β [17]. These findings, including those in the current study, showed that the phycocyanin-containing extract of spirulina is not toxic at dose <1000 µg/mL.

#### 3.1.2. Antioxidant Activity

In this research, DPPH radical scavenging activity was selected to evaluate the antioxidant activity of phycocyanin, as it is the simplest method and it is commercially available. DPPH is a stable free radical and receives an electron or hydrogen radical to become a stable diamagnetic molecule. The antioxidants contribute hydrogen or electron to reduce the stable radical DPPH to DPPH-H (non-radical form). The reduction can be observed from the changes of color from purple to yellow, and from the result of absorbance at 517 nm, the reduction capability of DPPH radical can be determined by the decrease in absorbance. Table 1 shows the antioxidant activity of phycocyanin based on the DPPH scavenging activity.

It can be observed from Table 1 that the DPPH scavenging activity increases with increasing phycocyanin concentration and was the highest at 625 µg/mL concentration with 67.90% scavenging activity. From the data obtained, the IC50 value was found to be at 14.22. In past research, Molyneux [18] explained that the IC50 parameter will show a lower value of IC50 when the antioxidant activity is higher, thus explaining why the IC50 value obtained for phycocyanin in this research was low compared to the high antioxidant activity. From the result shown, it can be said that phycocyanin is suitable to be incorporated as an active ingredient in a cosmetic patch, as the radical DPPH scavenging activity is good.

### 3.2. Optimization of Preparation Conditions

#### 3.2.1. Optimization of Phycocyanin/Alginate Ratio

**Figure 3:**
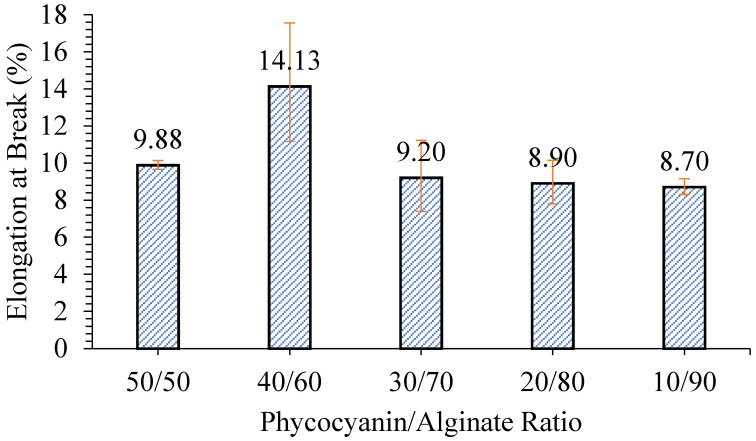
Elongation at break of patches with various phycocyanin/alginate ratios.

The test was further carried out on the optimization of preparation conditions for the patches using the one-factor-at-a-time (OFAT) method. Two responses were selected: elongation at break and releasing properties. The first test was done to study the effect of different phycocyanin/alginate ratios on the flexibility and releasing properties of the patch formed. It can be seen from Figure 3 that samples with a phycocyanin/alginate ratio of 90/10, 80/20, 70/30, and 60/40 were not provided. This was because when the ratio of phycocyanin was higher than alginate, alginate could not be dissolved and caused non-homogeneous films. The sample with a ratio of 40/60 phycocyanin/alginate exhibited the highest elongation at break (14.13%), while the other samples had almost similar elongation at break. Similar findings were also observed where the flexibility of the patch increased with increasing alginate content but then started to decrease when it reached the maximum alginate content [19].

**Figure 4:**
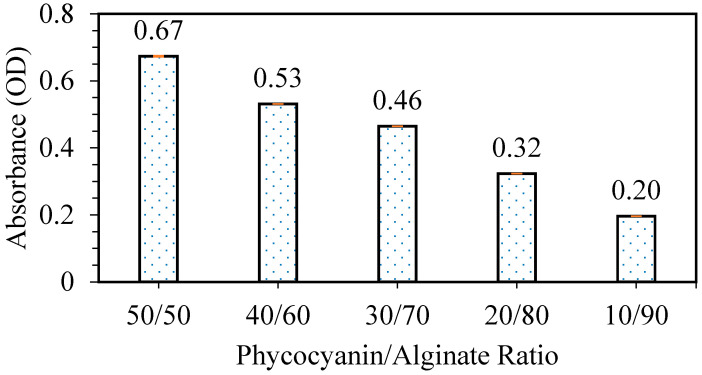
Releasing test of patches with various phycocyanin/alginate ratios.

Next, the releasing test was carried out in order to study the effect of different phycocyanin/alginate ratios on the releasing properties of the patches. From Figure 4, it can be observed that as the ratio of phycocyanin to alginate decreases (alginate content increases), the releasing capabilities of the patch decrease. This can be clearly explained by the decreasing content of phycocyanin in the patch, resulting in a higher amount of light passing through the solution dipped with the patch, hence the lower OD obtained [6].

The percentages of phycocyanin released (*w/w*%) are shown in Table 2. The concentration released was calculated based on a calibration curve equation of y = 0.2278x (R^2^ = 0.9), where y is the phycocyanin concentration (g/mL) and x is the absorbance unit (at 620 nm), and the values are obtained from Figure 4. The percentage released indicated that as the concentration of phycocyanin was reduced, the releasing was increased. Considering the elongation at break and releasing test of various ratios of phycocyanin/alginate, a ratio of 40/60 was chosen for the next optimization study (stirring time). The sample with the highest elongation at break was chosen, as the patch needs to be flexible with good releasing properties.

#### 3.2.2. Optimization of Stirring Time

**Figure 5:**
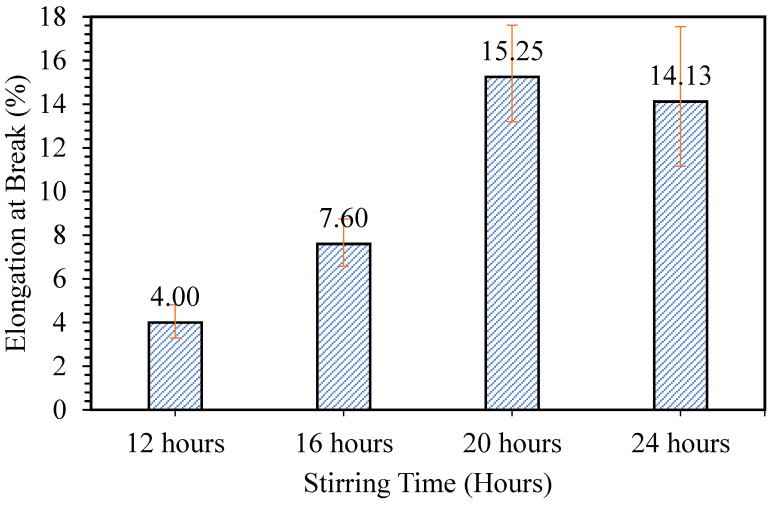
Elongation at break of patches prepared at different stirring times.

The second optimization step was done to study the effect of stirring time on the flexibility and releasing properties of the samples formed. Figure 5 shows the effect of stirring time on the flexibility of the patch formed. The sample prepared at 8 h was not tested, as the sample was non-homogeneous. It can be observed that the sample mixed for 20 h possesses the highest elongation at break, which is at 15.25%. It was assumed that as the stirring time increased, the mixture of phycocyanin/alginate would become more homogeneous, producing a better patch. However, for the sample with phycocyanin/alginate mixture mixed for 24 h, the elongation at break slightly decreased. This may have resulted from the longer stirring time, making the bonds break in the mixture of phycocyanin/alginate. Grasmeijer et al. [20] stated that prolonged mixing may cause detachment of mixed components.

**Figure 6:**
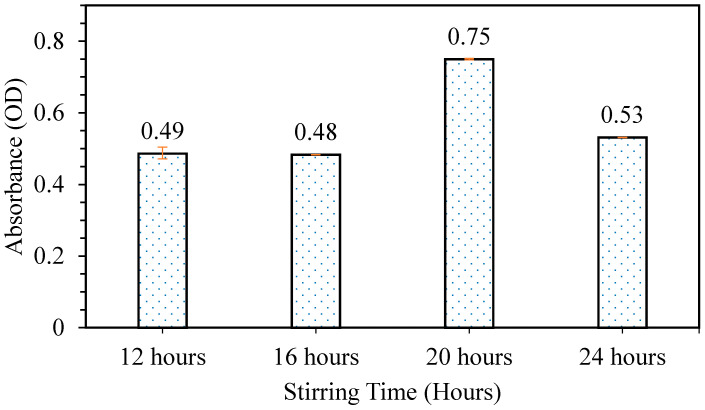
Releasing test of patches prepared at different stirring times (phycocyanin/alginate ratio at 40/60).

The releasing test was carried out to study the effect of stirring time on the releasing properties of patches formed. From Figure 6, it can be observed that the sample with phycocyanin/alginate mixture mixed for 20 h gave the highest releasing properties at 0.75. It can be assumed as previously explained for the flexibility test that, as the mixture of phycocyanin/alginate was mixed for an appropriate amount of time, the mixture became homogeneous, thus forming a patch with good properties. Based on the results obtained from the tensile test and releasing test, it was concluded that the best stirring time was at 20 h, as the patch produced had the best flexibility and releasing properties. The condition of a phycocyanin/alginate ratio of 40/60 that was stirred at 20 h was chosen for the next optimization study (mixing temperature).

#### 3.2.3. Optimization of Mixing Temperature

**Figure 7:**
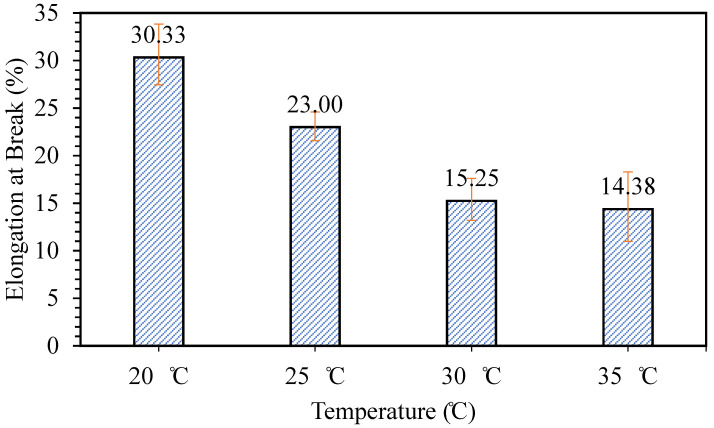
Elongation at break of patches prepared at different mixing temperatures.

The final test was done to study the effect of mixing temperature on the flexibility and releasing properties of the patch formed. Figure 7 presents the effect of temperature on the elongation at break of the patch formed. It can be observed that the sample prepared at mixing temperature 20 °C has the highest elongation at break at 30%. However, as the mixing temperature increases, the elongation at break of the samples prepared decreases. De Morais et al. [21] stated that phycocyanin is stable at a lower temperature, and the stability decreases as the temperature increases, especially above 40 °C. This could be the reason why the elongation at break of the samples decreases as the temperature increases, as the instability of phycocyanin at a higher temperature may affect the mechanical properties of samples formed.

**Figure 8:**
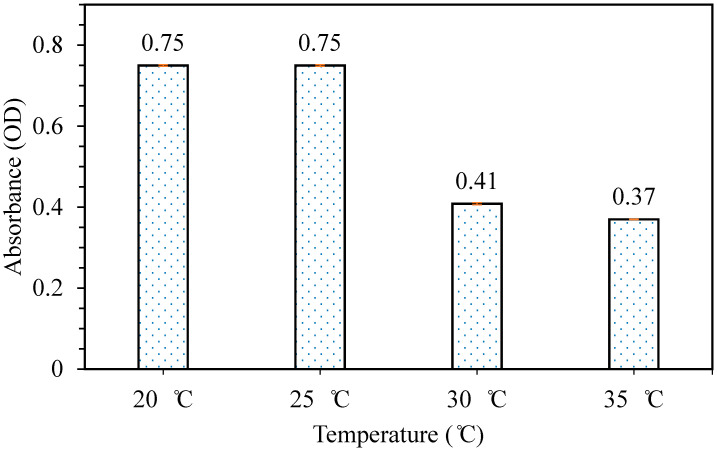
Releasing test of patches prepared at different mixing temperatures.

The releasing test was then carried out in order to study the effect of mixing temperature on the releasing properties of patches formed. Figure 8 shows the result obtained from the releasing test carried out. It can be observed that the releasing properties for samples prepared (40/60 phycocyanin/alginate ratio) at mixing temperatures 20 and 25 °C were the highest at 0.75. However, as the mixing temperature increased from 30 °C onwards, the releasing properties decreased. This may be tied to the results obtained from the elongation at break of the samples, because as a sample becomes more flexible, the releasing properties improve as well. Therefore, from the results obtained from the tensile test and releasing carried out, it was concluded that the sample prepared at mixing temperature 20 °C had the best flexibility and releasing properties. Based on the results obtained from optimizing the conditions, a phycocyanin/alginate ratio of 40/60, stirred for 20 h at 20 °C gave the best condition for flexibility and releasing properties.

### 3.3. Morphological Properties of PLA/Phycocyanin–Alginate Composite (Scanning Electron Microscopy (SEM))

**Figure 9:**
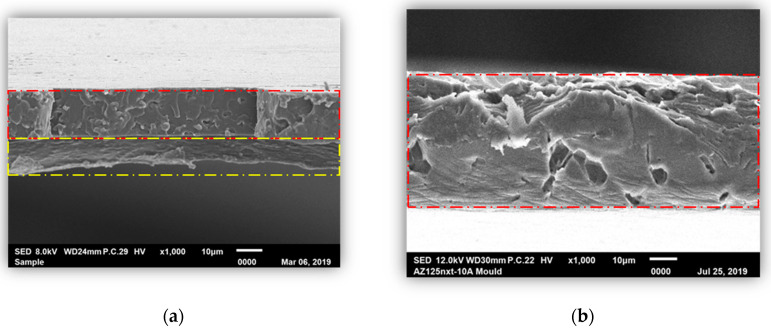
SEM micrographs of polylactic acid (PLA)/phycocyanin–alginate composite (**a**) before and (**b**) after the releasing test.

An SEM test was carried out in order to observe the morphological properties of the PLA/phycocyanin–alginate composite (sample selected from optimization study of preparation conditions), particularly for the sample before and after the releasing test. This was done to observe whether the active ingredient, phycocyanin, was released after the releasing test. Figure 9 shows SEM images taken of the sample before and after the releasing test. It can be identified that the upper layer (red dash line) was PLA, while the bottom layer (yellow dash line) was a phycocyanin/alginate layer. After the releasing test (Figure 9b), it can be observed that only one layer was observed, and it referred to the PLA layer. From this observation, we can assume that phycocyanin and alginate were released, as the second layer was not observed after the releasing test.

### 3.4. Attenuated Total Reflectance–Fourier Transform Infrared Spectroscopy (ATR–FTIR) of PLA/Phycocyanin–Alginate Composites

**Figure 10:**
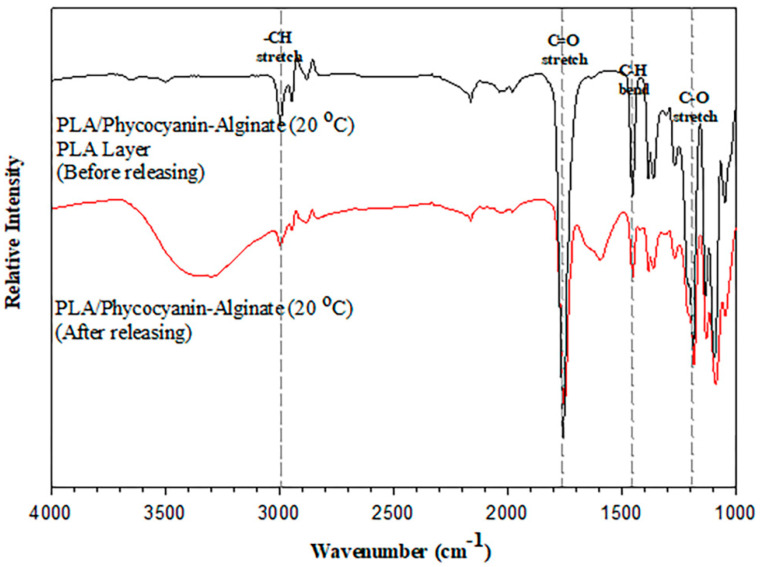
Attenuated total reflectance (ATR)–FTIR spectra for PLA/phycocyanin–alginate composites (20 °C) before and after the releasing test.

An ATR–FTIR test was conducted on the composite at optimized conditions, which were the PLA/phycocyanin–alginate composite prepared at a phycocyanin/alginate ratio of 40/60, stirred at 20 h and 20 °C. From Figure 10, it can be observed that there was no shift in absorption bands for the PLA/phycocyanin–alginate composite (20 °C) after the releasing test. Furthermore, this result was taken from the phycocyanin/alginate layer of the patch that had been released, and it can be seen that the characteristic of the absorption band was similar to the absorption band of the PLA layer of the composite before releasing. From this result, it can be assumed that phycocyanin and alginate had been fully released after the releasing test.

## 4. Conclusions

In this research, it was proven that the raw material used, phycocyanin, gave no cytotoxic effect to the skin cells and was considered safe to be used as an active ingredient for a cosmetic patch. From the process optimization results obtained, the PLA/phycocyanin–alginate composite with a phycocyanin/alginate ratio of 40/60 prepared for 20 h at 20 °C showed the potential to be used as a cosmetic patch, as it gave the best properties in terms of flexibility and provided fast release of active ingredients. Based on the IR spectra and SEM imaging, it can be assumed that phycocyanin and alginate were fully released after the releasing test. The PLA/phycocyanin–alginate composite was prepared in this research using a simple casting method, and it could be explored further to produce various patches using different active ingredients.

## Figures and Tables

**Table 1:** Antioxidant activity of the phycocyanin.

Phycocyanin Concentration (µg/mL)	DPPH Scavenging Activity (% Inhibition)
19.53	39.26
39.06	46.16
78.13	48.98
156.25	52.17
312.50	59.46
625.00	67.90

**Table 2:** Percentage of phycocyanin released.

Phycocyanin/Alginate Ratio	Percentage Release (*w/w*%)
50/50	30.52
40/60	30.18
30/70	34.93
20/80	36.45
10/90	45.56
